# Sperm Meiotic Segregation Analysis of Reciprocal Translocations Carriers: We Have Bigger FISH to Fry

**DOI:** 10.3390/ijms24043664

**Published:** 2023-02-11

**Authors:** Edgar Del Llano, Aurore Perrin, Frédéric Morel, Françoise Devillard, Radu Harbuz, Véronique Satre, Florence Amblard, Marie Bidart, Sylviane Hennebicq, Sophie Brouillet, Pierre F. Ray, Charles Coutton, Guillaume Martinez

**Affiliations:** 1 Genetic Epigenetic and Therapies of Infertility, Institute for Advanced Biosciences INSERM U1209, CNRS UMR5309, 38000 Grenoble, France; 2 Department of Medical Genetics and Reproductive Biology, Brest University Regional Hospital, 29200 Brest, France; 3 Inserm, Université de Bretagne Occidentale, EFS, UMR 1078, GGB, 29200 Brest, France; 4 UM de Génétique Chromosomique, Hôpital Couple-Enfant, Centre Hospitalier Universitaire de Grenoble, 38000 Grenoble, France; 5 Centre Clinique et Biologique d’Assistance Médicale à la Procréation, Hôpital Couple-Enfant, Centre Hospitalier Universitaire de Grenoble, 38000 Grenoble, France; 6 DEFE, Université de Montpellier, INSERM 1203, Hôpital Arnaud de Villeneuve, CHU de Montpellier, IRMB, 80 Avenue Augustin Fliche, CEDEX 05, 34295 Montpellier, France

**Keywords:** reciprocal translocation, meiotic segregation pattern, FISH, sperm chromosomes, semen analysis, male infertility, preimplantation genetic testing

## Abstract

Reciprocal translocation (RT) carriers produce a proportion of unbalanced gametes that expose them to a higher risk of infertility, recurrent miscarriage, and fetus or children with congenital anomalies and developmental delay. To reduce these risks, RT carriers can benefit from prenatal diagnosis (PND) or preimplantation genetic diagnosis (PGD). Sperm fluorescence in situ hybridization (spermFISH) has been used for decades to investigate the sperm meiotic segregation of RT carriers, but a recent report indicates a very low correlation between spermFISH and PGD outcomes, raising the question of the usefulness of spermFISH for these patients. To address this point, we report here the meiotic segregation of 41 RT carriers, the largest cohort reported to date, and conduct a review of the literature to investigate global segregation rates and look for factors that may or may not influence them. We confirm that the involvement of acrocentric chromosomes in the translocation leads to more unbalanced gamete proportions, in contrast to sperm parameters or patient age. In view of the dispersion of balanced sperm rates, we conclude that routine implementation of spermFISH is not beneficial for RT carriers.

## 1. Introduction

Reciprocal translocations (RTs), resulting from an exchange of terminal segments between two non-homologous chromosomes, are the most common structural chromosome rearrangements with an estimated prevalence of 0.14% in newborns [1]. Balanced carriers of RTs are phenotypically normal as their genome contains the normal amount of chromosomal information despite being rearranged on different chromosomes. However, meiotic gametes production by RT carriers may lead to gametes with unbalanced chromosomal content and thus exposes them to higher risks of infertility, recurrent miscarriage, and fetus or children with congenital anomalies and developmental delay [2,3]. Therefore, the prevalence of RT increases to 0.6% in the infertile population, 1.2% in azoospermic men, 2.7% in couples with repeated implantation failures, and up to 6.9% in couples with recurrent miscarriages [4,5,6], establishing RTs as a major contributor to infertility.

Unbalanced gametes are produced in RT carriers during meiosis depending on how the segregation of the derivative chromosomes and their normal homologous takes place. During meiosis, translocated chromosomes and their homologous can segregate according to 5 different patterns: alternate, adjacent−1, adjacent−2, 3:1 (exchange and tertiary), and 4:0 segregations [7]. Alternate segregation produces balanced gametes by segregating the normal chromosomes in one cell and the two derivatives in another. In adjacent−1 both non-homologous and in adjacent−2 both homologous centromeres segregate together. In 3:1, 3 out of 4 chromosomes segregate together and in 4:0 segregations all 4 chromosomes are pulled into one pole. There are up to 32 different possible products of these gametes after the aforementioned combinations and possible chromosome recombinations and only 2 of them possess balanced chromosomal content [7,8].

Depending on each country’s law and availability, RT carriers could have access to two main possibilities to minimize the risk of unbalanced offspring when using their own gametes. Prenatal diagnosis (PND) on chorionic villus sampling or amniocentesis can provide information on the fetus’s genetic status, but since the diagnosis is obtained post-implantation during an evolutive pregnancy, it does not reduce the risk of miscarriage [9]. Preimplantation genetic diagnosis (PGD) on embryo biopsy, with or without screening for other chromosome aneuploidy, gives information about the viability of the embryos before implantation and therefore reduces the risk of spontaneous pregnancy termination [9,10].

Sperm fluorescence in situ hybridization or spermFISH has been used for several decades to investigate the number of balanced gametes in semen and the proportions of the segregation modes in sperm from numerous species [11]. The most important contribution of spermFISH would be its potential ability to be used as a predictor of the proportions of balanced embryos obtained after PGD fertilization. Unfortunately, recent studies on the subject find no correlations, or only a very weak trend, between balanced rates of sperm and embryos [3,9].

Several studies tried to link the greater risk of chromosomal unbalance with the age and sex of the carriers [12,13,14], the sperm parameters [15,16], or the type of chromosome involved in the translocation [10,17]. Because of the paucity of patients in many of them (mean of 3.6 patients per study with only 2 including more than 12 patients [15,18]), these studies obtained conflicting results. Wider cohorts and compiled analysis of all patients from the literature are needed to reach a clearer vision of these potential correlations.

In the present study, we report data from 41 RT male carriers, the biggest cohort reported so far, and conducted a review of all 318 patients from the literature. We report the results of their meiotic segregation rate for each possibility and address the factors that may or may not influence these rates. We also discuss the key question of whether spermFISH is still a useful tool for RT carriers.

## 2. Results

### 2.1. RT Carriers Produce between 20 and 90% of Balanced Sperm

**Figure 1:**
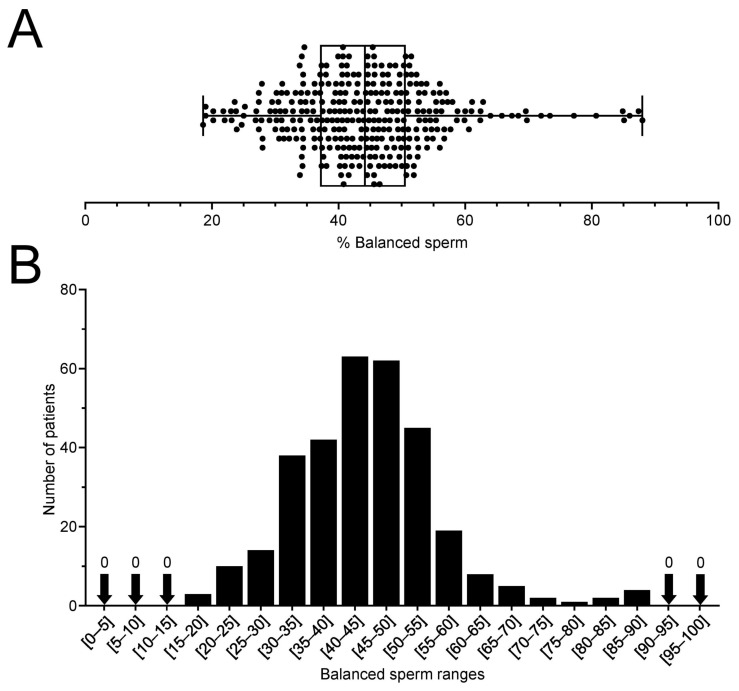
Balanced sperm rates of 318 reciprocal translocation carriers. (**A**) Box-and-whisker plot of balanced sperm rates of all patients with each data point displayed as a dot. (**B**) Histogram of the number of patients according to 5% ranges of balanced sperm.

Segregation rates of our 41 patients are provided in Table 1 with balanced sperm rates ranging from 27.83 to 69.75%. The average rate of balanced gametes among all 318 patients (our patients and 277 literature patients) is 44.26% ± 8.64% (min 18.6–max 88). Balanced rates dispersion (Figure 1A,B) reveals that most (85%) of the RT carriers display a 30–60% rate, with only 3 patients (0.9%) producing less than 20% of balanced sperm, although with a rate only slightly reduced as it remained between 18–20%. On the other side of the spectrum, only 6 patients (1.9%) of carriers surpassed the ≥80% of balanced sperm. Interestingly, no carriers displayed extreme segregation rates presenting ≤15% or ≥90% of balanced rates. 

### 2.2. Abnormal Sperm Parameters Do Not Correlate with Unbalanced Chromosome Content

**Figure 2:**
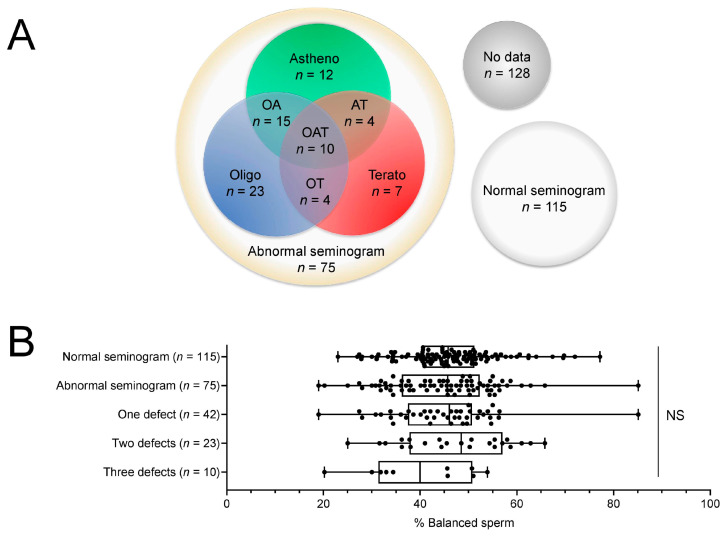
Relationship between balanced sperm rates and the number of sperm parameter defects. (**A**) Graphical representation of the different patient populations according to their sperm parameters. (**B**) Box-and-whisker plot of balanced sperm rates according to individual seminogram results with each data point displayed as a dot. The boxes represent the interquartile range with the median as the box centerline and 1st and 3rd quartile at the low and high box lines. The whiskers represent the minimum and maximum values. n is the number of patients, NS—not significant. Corresponding statistical data can be found in the Supplementary Materials Table S1.

We looked for possible correlations between sperm parameters and balanced chromosome rates of all RT carriers. Out of 318 patients, 115 had a normal seminogram, 75 an abnormal seminogram (including asthenozoospermia, oligozoospermia, and/or teratozoospermia) and no data were available from 128 (Figure 2A). The presence of a normal or abnormal seminogram (without taking into account the different percentages of each abnormality) did not impact the balanced sperm rates (mean ± SD 46.22 ± 7.32% versus 44.86 ± 8.72%, *p* = 0.37, Supplementary Materials Table S1). The number of defects reported in the seminogram (quantity, motility, and morphology) also has no impact on segregation rates, although we observed a non-significant decrease when all three defects were present (Figure 2B, Supplementary Materials Table S1).

**Figure 3:**
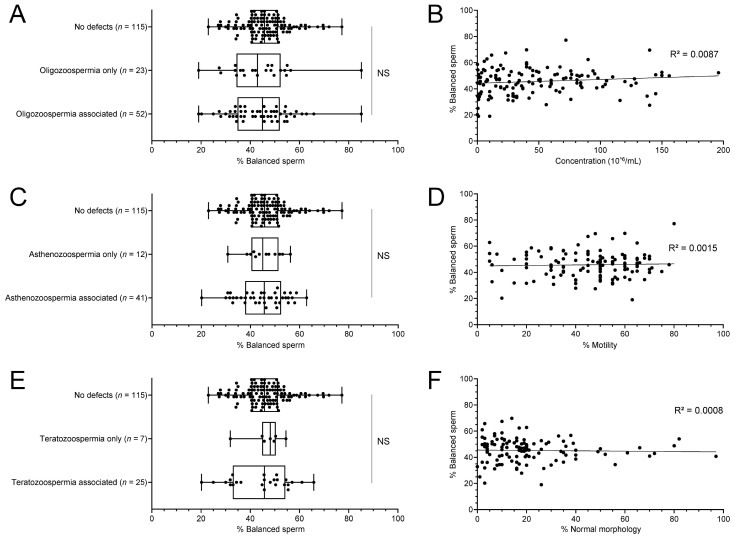
No correlation observed between balanced sperm rates and sperm parameters. Concentration (**A**,**B**), motility (**C**,**D**), and morphology (**E**,**F**) parameters are not correlated with the rate of balanced sperm in RT carriers, whether each parameter is considered alone or associated with others. NS—not significant. Corresponding statistical data can be found in Supplementary Materials Tables S2–S4.

Furthermore, we looked for correlations by investigating the sperm parameters one by one. Again, we did not uncover a significant correlation between sperm parameters and balanced sperm rates (Figure 3, Supplementary Materials Tables S2–S4), whether considered alone or in combination with another defect.

### 2.3. RT Carriers Display Less Balanced Sperm When an Acrocentric Chromosome Is Involved in the Translocation, Especially Chromosome 22

**Figure 4:**
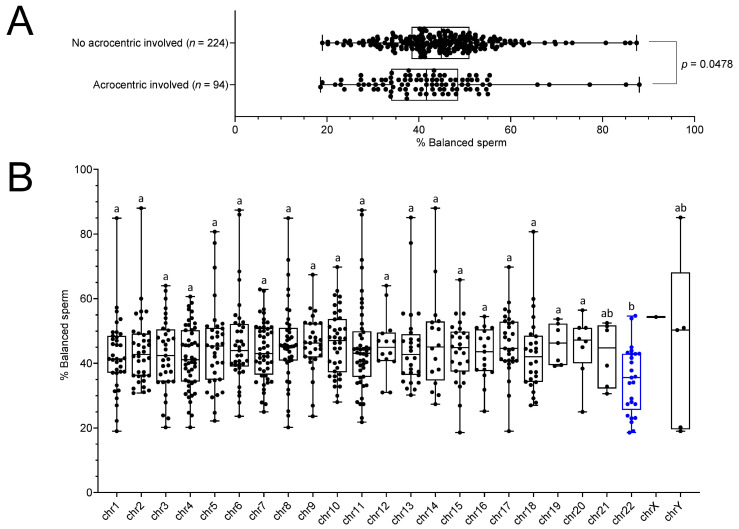
Acrocentric chromosomes, especially 22, reduce the balanced sperm rates. The rate of balanced cells is reduced (**A**) when an acrocentric chromosome is involved in the translocation, (**B**) especially chromosome 22 (blue) whose presence is associated with higher rates of imbalance. Each chromosome was compared individually with all other chromosome one by one. For each chromosome, plots sharing different small letters represent statistically significantly differences between the groups (*p* < 0.05), and plots with a common letter do not present statistically significantly differences between the groups (*p* > 0.05). Corresponding statistical data can be found in the Supplementary Materials Tables S5 and S6.

We investigate whether the involvement of specific chromosomes in the translocation could induce a deleterious or beneficial effect on segregation rates. Interestingly, our data indicate that translocations involving acrocentric chromosomes have more risk of segregating in ways leading to unbalance gametes compared with the rest of the chromosomes (Figure 4A, Supplementary Materials Table S5). Moreover, we investigated each chromosome individually and detected a reduction in balanced rates in carriers with translocations involving chromosome number 22 when compared with the other chromosomes (Figure 4B, Supplementary Materials Table S6). As t(11;22) is the most frequent RT and is known for its tendency to favor low balanced gamete rates [19], it raised the question of whether this specific translocation alone could be responsible for the observed effect. We performed statistical analysis with only or with exclusion of t(11;22) translocations and this did not affect the results (Supplementary Materials Table S5), confirming that the deleterious effect is indeed associated with chromosome 22 itself and not with t(11;22).

## 3. Discussion

The foremost point of this work was to examine whether spermFISH is a tool worthy to be used regularly for genetic counseling in RT carriers [3,9]. As some studies observed only a very weak trend between sperm balanced rates of RT carriers and embryos produced by PGD [3,9], only extreme values could be considered of interest. A patient with less than 10% of balanced gametes would suggest the production of a very low number of balanced embryos in PGD. Therefore, there would be the need to initially obtain a large number of embryos for analysis, which is not always a possibility, depending on the other clinical data of the patient and his partner. In this situation, the low chances of success of PGD and the analysis of the benefit/risk balance should be discussed with the couple. These same considerations must be taken into account for RT carriers whose gametes are more than 90% balanced, given the small difference they would have with normo-fertile men. None of the patients in our cohort or in the literature had any of these extreme values with more than 85% of patients having levels in the 30–60 range and more than 97% of patients in the 20–80 range, which would leave less than 3% of patients in a potential gray zone. As PGD appears to benefit all RT carriers indiscriminately and spermFISH appears to be neither a necessary prerequisite nor a useful tool for them, we do not encourage routine implementation of spermFISH for RT carriers. It should be noted that these results reflect our current knowledge and that additional studies incorporating patient results for preimplantation genetic testing for chromosome structural rearrangements could greatly influence the conclusions reached in this paper.

It has been previously well described that sperm parameters are commonly altered in RT carriers [15,16,20], raising the question of whether these alterations could be correlated with balanced gamete rates. Our results show no differences in balanced gamete rates between patients with a normal or impaired seminogram, and furthermore none of the major sperm parameters are correlated either. It should be noted that we also did not observe any influence of the patient’s age on segregation rates (Supplementary Materials Figure S1). Therefore, all these parameters cannot be considered to predict balanced gamete rates of carriers. Other parameters could be interesting candidates and would have the merit to be investigated, such as body mass index, toxic consumption, and toxic exposure.

Several groups reported a lower frequency of alternate segregation in RT carriers when acrocentric chromosomes are involved in the translocation compared with non-acrocentric [10,13,14,21]. In our analysis, this effect is confirmed as carriers with translocations involving acrocentric chromosomes have a modest but significant reduction in balanced sperm. Furthermore, our data confirm another previously identified link between unbalanced sperm and the chromosome 22 specifically [13,17,22]. This information should be delivered to patients before processing PGD.

It is necessary to underline that although we conclude here that there is no benefit in performing meiotic segregation analysis for RT carriers in routine implementation, spermFISH remains a valuable tool for all other structural rearrangements that may lead to either very high or low balanced gamete rates, such as complex chromosomal rearrangements (see review in [23]) or inversions [24,25,26,27,28].

FISH techniques, in the broadest sense, remain a staple of genetic diagnosis within genetic laboratories despite the rise of genomic technologies [29], and continue to be improved as evidenced by the constant emergence of new devices [30,31,32] and techniques such as smRNA-FISH, oligopaint-FISH, or CAS-FISH. Novel FISH techniques allow us to visualize the genome at resolutions never achieved before. Oligopaint technology [33,34] that generates single-stranded oligonucleotide probes can be used in combination with high-resolution microscopy techniques (OligoSTORM-FISH with stochastic optical reconstruction microscopy and dnaPAINT-FISH with DNA-based dot accumulation for nanoscale topography imaging [35,36,37,38]) and should allow us to further explore the DNA of gametes from rearrangement carriers. Moreover, the advent of GOLD-FISH (genome oligopaint via local denaturation) and RASER-FISH (resolution after single-strand exonuclease resection) now enables us to avoid the harsh decondensation step required in conventional FISH and thus study intact chromatin architecture in spermatozoa [39]. In the end, these developments could become or give birth to regular tools for research, cytogenetics, or fertility centers and improve patient diagnostics and management.

## 4. Materials and Methods

### 4.1. Patients

Forty-one male patients carrying a RT were included in this retrospective cohort study. They consulted in the genetic and procreation department of the university hospital of Grenoble or in the department of medical genetics and reproductive biology of Brest university hospital between 2006 and 2021. Karyotyping on blood cells identified all translocations and their breakpoints. All spermFISH were performed as a routine test ruled by a signed informed genetic consent in accordance with local protocols and the principles of the Declaration of Helsinki. All reagents were purchased from Sigma-Aldrich unless specified otherwise.

### 4.2. Literature Search

**Figure 5:**
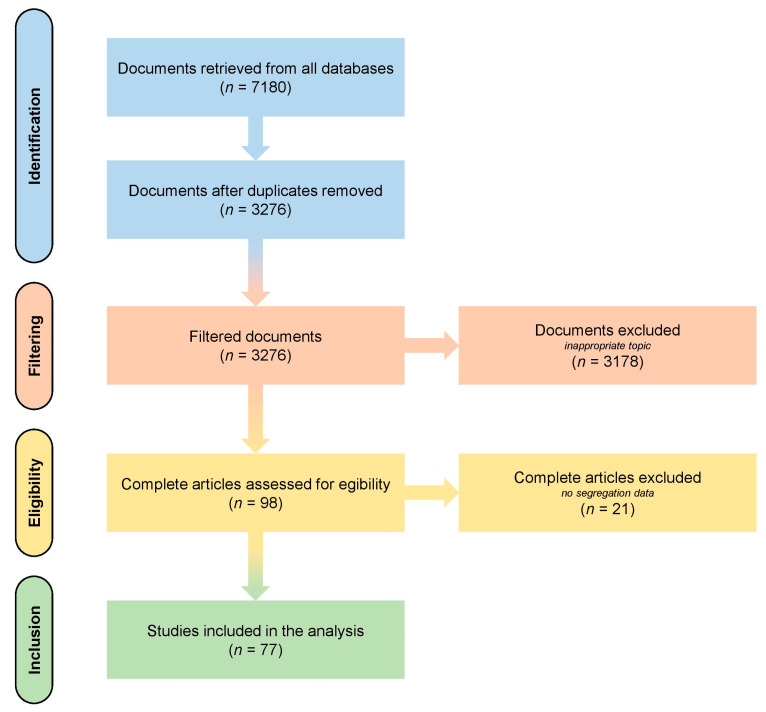
Flow diagram of the studies. The studies included in the analysis are listed in the Supplementary Materials Table S7.

The PubMed/MEDLINE database was screened from inception to September 2022 using combinations of MESH terms reciprocal translocation/meiotic segregation/spermFISH. Additional studies were retrieved from the reference lists of full articles. All extracted articles were reviewed and after excluding all articles not presenting sperm segregation rates data of reciprocal translocation carriers, we included 277 patients from 77 studies with our 41 patients (Figure 5). All selected references are presented in the Supplementary Materials Table S7. The following data were extracted from each reference when available: publication year, study authors, title, patient karyotypes, segregation rates, sperm parameters (concentration, motility, and morphology), and age.

### 4.3. Sperm Technique

Semen samples were collected by masturbation in a sterile container and maintained at 37 °C for 30 min to allow liquefaction. We evaluated sperm concentration, motility, and morphology according to World Health Organization criteria for human semen analysis [40].

Each sperm sample was treated as previously described [41]. Briefly, samples were washed in PBS 1×, fixed in methanol/acetic acid (3:1, *v/v*) solution, spread on slides, decondensed in NaOH 1 M solution, and dehydrated in ethanol solutions. Each sample was co-denatured with its specific probes mix for 2 min at 75 °C and then hybridized for 18 h in a HYBrite^®^ system (Abbott Laboratories, Chicago, IL, USA). Slides were then washed according to manufacturer specifications and then mounted with DAPI II (Abbott Laboratories) to counterstain sperm nuclei. All probes and mixes used for each patient are presented in the Supplementary Materials Table S8.

### 4.4. FISH Scoring

Scoring was performed half manually and half with a METAFER Metasystems^®^ device, previously validated for spermFISH analysis [42]. Experienced technicians or engineers manually checked the galleries of images provided by the machine. Strict criteria were applied: spermatozoa had to be intact with clearly defined border, no swelling, and non-overlapping. To be eligible, hybridization signals must be clearly delineated, positioned inside the head, similar in size and brightness, and separated by at least the size of a signal. Segregation patterns were determined according to the signal observed. A minimum of 1000 cells were counted per patient (mean = 1782, min = 1000, max = 2789).

### 4.5. Statistical Analysis

Data were treated with R software (version number 2.14.1). A probability value of less than 0.05 was considered to be statistically significant.

Box-and-whisker plots display each data point as dots. The boxes represent the interquartile range with the median as the box centerline and 1st and 3rd quartile at the low and high box lines. The whiskers represent the minimum and maximum values.

Scatter plot display each data point as dots and present linear regression with R indicated on the graph.

## Figures and Tables

**Table 1:** Reciprocal translocation carriers: age, translocation, meiotic segregation, number of cells counted, PMRS (risk of having a child with a polymalformative mental/retardation syndrome), semen parameters and reproductive history. 3:1 ech: 3:1 exchange; 3:1 ter: 3:1 tertiary.

Patient	Age	Translocation	Segregation (%)						Number of Counted Cells	PMRS (%)	Sperm Parameters			Reproductive History
			Alternate	Adjacent1	Adjacent2	3:1 ech	3:1 ter	Others			Concentration (×10^6^/mL)	Motility (%)	Morphology (% Normal)	
P01	31	(1;4) (p21;q13)	31.36	14.40	14.05	9.76	23.75	7.14	1722	0.11	25	40	27	Spontaneous abortions/Repeated miscarriages
P02	35	(1;6) (q42;p22)	52.73	25.39	5.47	10.16	6.25	0.00	1024	21.15	50	60	26	Spontaneous abortions/Repeated miscarriages
P03	49	(1;11) (p34;q24)	49.67	36.14	1.74	7.47	4.98	0.00	1606	22.79	90	45	2	Infertility
P04	-	(1;11) (q12;q24)	57.23	26.42	0.00	5.03	10.69	0.63	1590	0.49	nc	nc	nc	Familial study
P05	-	(2;4) (q24;p15.1)	42.19	29.86	10.68	9.32	7.95	0.00	2190	2.71	52	70	52	Infertility
P06	31	(2;7) (p25;p15.1)	36.19	30.08	2.33	12.30	15.40	3.70	2487	27.06	1.8	60	2	Infertility
P07	33	(2;10) (q21;q24)	36.00	23.00	7.00	21.00	13.00	0.00	1000	0.60	2.6	50	4	Infertility
P08	28	(2;12) (q14.2;q15)	46.99	33.11	6.76	3.71	3.63	5.80	2588	0.23	8	60	19	Infertility
P09	36	(3;11) (p24;p12)	62.43	21.27	6.91	5.80	3.59	0.00	1448	10.44	90	65	17	Spontaneous abortions/Repeated miscarriages
P10	35	(3;12) (q13.1;q13)	50.19	27.63	13.62	5.45	3.11	0.00	1028	0.74	150	70	18	Infertility
P11	30	(4;8) (q31.1;p21)	47.14	31.31	3.70	12.79	5.05	0.00	1188	10.34	15.6	65	17	Infertility
P12	29	(4;10) (q21;q24)	37.50	27.00	7.25	13.25	7.25	7.75	1600	1.29	67	50	32	Spontaneous abortions/Repeated miscarriages
P13	38	(4;14) (p14;q32)	33.86	15.91	4.77	14.09	31.36	0.00	1760	31.54	1.6	40	8	Infertility
P14	29	(4;17) (p16.1;q21.3)	34.42	20.41	14.21	14.01	14.92	5.58	1970	17.86	10	55	10	Spontaneous abortions/Repeated miscarriages
P15	-	(4;21) (q28;q21)	50.25	35.47	4.43	2.96	5.42	1.48	1218	-	15	20	6	Spontaneous abortions/Repeated miscarriages
P16	26	(5;18) (p15.1;q12.3)	46.08	21.97	21.46	4.83	5.34	0.31	1966	14.58	105	65	26	Familial study
P17	29	(6;12) (p25;p13)	46.83	39.98	1.90	5.45	3.90	1.95	2001	32.24	50	60	18	Infertility
P18	36	(6;15) (p12;p13)	65.80	23.42	0.74	4.09	4.83	1.12	1614	29.03	11.6	55	3	Infertility
P19	31	(6;15) (q12;q21)	39.93	37.92	5.37	9.73	7.05	0.00	1788	4.29	22	70	23	Spontaneous abortions/Repeated miscarriages
P20	30	(6;18) (p21.1;q23)	40.51	30.55	9.15	7.94	8.83	4.35	2481	18.31	37	50	16	Familial study
P21	28	(6;18) (p21.1;q23)	27.83	27.50	6.32	8.06	19.44	10.85	1502	18.31	56	40	18	Familial study
P22	32	(7;9) (p14;q21)	42.11	23.98	9.65	13.45	10.82	0.00	1710	-	15	60	5	Infertility
P23	36	(8;16) (p12;q23)	31.84	23.38	3.48	21.39	19.90	0.00	1005	16.35	1	15	3	Infertility
P24	34	(8;16) (p12;q23)	45.32	31.72	7.85	7.25	7.85	0.00	1655	16.35	42	45	17	Familial study
P25	43	(8;20) (p12;q12)	56.41	13.46	23.72	3.21	3.21	0.00	1560	12.17	12	35	7	Spontaneous abortions/Repeated miscarriages
P26	30	(9;11) (p24;q23)	42.49	35.42	6.67	4.55	5.09	5.77	2789	25.74	84	50	12	Infertility
P27	56	(9;17) (p22;q23)	52.31	27.17	2.31	11.27	6.94	0.00	1730	18.74	3.5	50	13	Infertility
P28	43	(9;17) (q22;q21)	41.60	30.47	2.59	13.49	7.60	4.24	1697	5.40	102	30	20	Infertility
P29	34	(10;17) (q11.2;q25)	44.63	27.48	10.03	9.52	7.52	2.10	1954	14.58	33	65	19	Familial study
P30	29	(10;17) (p15;q12)	69.75	11.37	11.49	2.70	3.52	1.17	1706	4.86	40	60	14	Spontaneous abortions/Repeated miscarriages
P31	30	(13;15) (q32;q22)	33.95	34.15	4.86	10.88	11.43	4.72	1461	9.65	0.9	50	20	Spontaneous abortions/Repeated miscarriages
P32	40	(13;18) (q14;q22)	43.91	34.51	7.76	5.70	4.62	3.49	1947	17.35	45	35	4	Infertility
P33	-	(14;17) (q22;p11.2)	53.07	20.11	8.38	10.61	7.82	0.00	1790	11.44	27.5	20	11	Infertility
P34	43	(8;16) (p22;p12)	43.61	21.93	29.07	0.80	4.01	0.50	2002	-	60	20	35	Infertility
P35	41	(2;13) (q33;q14)	41.65	45.90	3.27	2.58	2.58	4.06	2019	-	24	42	36	Infertility
P36	32	(8;19) (p23;p11)	53.70	29.26	9.65	3.62	1.46	1.71	1989	-	76	59	8	Infertility
P37	32	(12;15) (q14;q25)	43.18	37.83	8.88	3.19	2.31	4.62	2038	-	31.3	43	25	Spontaneous abortions/Repeated miscarriages
P38	44	(8;20) (q24.3;q11)	47.60	41.95	4.57	1.73	0.70	2.61	2145	-	127.7	49	17	Infertility
P39	34	(4;5) (q35;q22)	53.40	44.90	0.35	0.20	0.35	0.45	2025	-	48.1	68	19	Spontaneous abortions/Repeated miscarriages
P40	36	(1;2) (p22;p14)	49.66	24.54	12.68	2.93	7.93	1.82	2082	-	11	56	7	Infertility
P41	34	(2;4) (p13;q27)	36.23	42.31	11.32	3.10	2.25	4.80	2004	-	145	56	35	Infertility

## Data Availability

All data generated or analyzed during this study are included in the manuscript and its Supplementary Materials.

## Supplementary Materials

The following supporting information can be downloaded at: https://www.mdpi.com/article/10.3390/ijms24043664/s1.
