# Supplementary material for: Sperm Meiotic Segregation Analysis of Reciprocal Translocations Carriers: We Have Bigger FISH to Fry
Source: Int J Mol Sci. 2023 Feb 11;24(4):3664. doi: 10.3390/ijms24043664 (PMC9965694; doi:10.3390/ijms24043664)
Supplement: Supplementary file 1 [file ijms-24-03664-s001.zip › ijms-2140812-supplementary.pdf]

Supplemental Table S1. Statistical data associated to the Student *t*-test performed in Figure 2B. DoF = Degrees of Freedom ; CI = Confidence Interval.

Groups	t-Value	DoF	*p*-Value	95% CI
Normozoospermia VS Abnormal seminogram	0.8875	188	0.3759	-1.661043 / 4.378443
Normozoospermia VS Only one defect	0.83149	155	0.407	-2.075214 / 5.092099
Normozoospermia VS Two defects	-0.51997	136	0.6039	-5.701207 / 3.327304
Normozoospermia VS Three defects	1.614	126	0.109	-1.048219 / 10.318699
Abnormal seminogram VS Only one defect	0.070346	115	0.944	-4.066686 / 4.366170
Abnormal seminogram VS Two one defect	-0.95854	96	0.3402	-7.817313 / 2.726009
Abnormal seminogram VS Three one defect	1.3937	83	0.1671	-2.231918 / 12.684084
Only one defect VS Two defects	-0.94521	63	0.3482	-8.393912 / 3.003124
Only one defect VS Three defects	1.3178	50	0.1936	-2.66069 / 12.81337
Two defects VS Three defects	1.8336	31	0.07633	-0.872880 / 16.416350

Supplemental Table S2. Statistical data associated to the Student *t*-test performed in Figure 3A. DoF = Degrees of Freedom ; CI = Confidence Interval.

Groups	t-Value	DoF	*p*-Value	95% CI
Normozoospermia VS Oligozoospermia alone	0.9473	136	0.3451	-2.453779 / 6.966729
Normozoospermia VS Oligozoospermia associated	1.1516	165	0.2511	-1.458752 / 5.542268
Oligozoospermia alone VS associated	-0.0677	73	0.9462	-6.532020 / 6.102587

Supplemental Table S3. Statistical data associated to the Student *t*-test performed in Figure 3C. DoF = Degrees of Freedom ; CI = Confidence Interval.

Groups	t-Value	DoF	*p*-Value	95% CI
Normozoospermia VS Asthenozoospermia alone	0.37098	125	0.7113	-4.657645 / 6.806601
Normozoospermia VS Asthenozoospermia associated	0.75309	154	0.4525	-2.159768 / 4.820923
Asthenozoospermia a alone VS associated	0.08537	51	0.9323	-5.766426 / 6.278624

Supplemental Table S4. Statistical data associated to the Student *t*-test performed in Figure 3E. DoF = Degrees of Freedom ; CI = Confidence Interval.

Groups	t-Value	DoF	*p*-Value	95% CI
Normozoospermia VS Teratozoospermia alone	-0.054772	120	0.9564	-7.631711 / 7.220834
Normozoospermia VS Teratozoospermia associated	0.81328	138	0.4175	-2.609099 / 6.254958
Teratozoospermia alone VS associated	0.42631	30	0.6729	-7.688594 / 11.745330

Supplemental Table S5. Statistical data associated to the Student *t*-test performed in Figure 4A. DoF = Degrees of Freedom ; CI = Confidence Interval.

Groups	t-Value	DoF	*p*-Value	95% CI
No acrocentric VS Acrocentric	1.9869	316	0.0478	0.027529 / 5.621170

Supplemental Table S6. Statistical data associated to the Student *t*-test performed in Figure 4B. To limit the size of the table, only significant results are presented. DoF = Degrees of Freedom ; CI = Confidence Interval.

Groups	t-Value	DoF	*p*-Value	95% CI
Chr1 VS Chr22	-2.6718	58	0.009777	-13.59633 / -1.94949
Chr2 VS Chr22	-3.3077	59	0.001606	-14.78053 / -3.63820
Chr3 VS Chr22	-2.7754	56	0.00748	-13.75338 / -2.22246
Chr4 VS Chr22	-2.8528	71	0.00567	-12.05256 / -2.13574
Chr5 VS Chr22	-3.2513	57	0.00193	-16.73108 / -3.97696
Chr6 VS Chr22	-3.6179	60	0.00061	-18.08700 / -5.20771
Chr7 VS Chr22	-3.8186	71	0.00028	-13.58467 / -4.26447
Chr8 VS Chr22	-3.9101	65	0.00022	-17.25789 / -5.58869
Chr9 VS Chr22	-4.2775	52	8.117e-05	-16.30628 / -5.89240
Chr10 VS Chr22	-4.3853	61	4.658e-05	-16.6958 / -6.23824
Chr11 VS Chr22	-3.1415	69	0.00247	-16.54957 / -3.69430
Chr12 VS Chr22	-3.1829	37	0.00295	-17.38552 / -3.86059
Chr13 VS Chr22	-3.1487	51	0.00274	-16.47396 / -3.64572
Chr14 VS Chr22	-2.9701	39	0.00507	-20.11488 / -3.81676
Chr15 VS Chr22	-3.1832	49	0.00253	-14.67763 / -3.31737
Chr16 VS Chr22	-2.7335	40	0.00928	-14.15925 / -2.12173
Chr17 VS Chr22	-4.1214	52	0.00013	-16.75768 / -5.78306
Chr18 VS Chr22	-2.578	49	0.01299	-14.26990 / -1.76819
Chr19 VS Chr22	-2.7136	30	0.01092	-19.67094 / -2.77659
Chr 20 VS Chr22	-2.4952	31	0.01812	-18.80326 / -1.88963

Supplemental Table S7. References included in this study.

Year of Publication	Authors	Number of Patients	% Alternate Range	References [1]
1983	Balkan & Martin	2	68,4-80,7	10.1007/BF00274760
1984	Martin et al.	1	23,1	10.1111/j.1399-0004.1984.tb02004.x
1986	Brandriff et al.	2	37,1-37,3	PMID: 3946422
1986	Burns et al.	2	27,4-69,6	PMID: 3728467
1988	Martin	1	40,4	10.1159/000132504
1988	Templado et al.	1	73,4	10.1007/BF00291704
1989	Pellestor et al.	4	34,2-77,2	PMID: 2818710
1990	Martin et al.	1	46,8	10.1093/oxfordjournals.humrep.a137153
1990	Martin et al.	3	43,3-52,7	10.1007/BF00205168
1990	Templado et al.	1	40,9	10.1007/BF00208932
1992	Estop et al.	2	39-46	10.1007/BF00194315
1992	Jenderny	2	34,2-56	10.1007/BF00210768
1992	Martin	2	39,5-45,6	10.1159/000133285
1992	Spriggs et al.	1	38,6	10.1007/BF00215680
1993	Martin & Hulten	3	34,1-47,7	10.1111/j.1601-5223.1993.00165.x
1994	Benkhalifa et al.	2	27,9-49,3	Rev Fr Gynecol Obstet (1994)
1994	Martin	1	55,4	10.1093/oxfordjournals.humrep.a138739
1994	Spriggs et Martin	1	33,2	10.1002/mrd.1080380303
1995	Estop et al.	2	40,2-48,0	10.1159/000133997
1995	Martin & Spriggs	1	46,9	10.1111/j.1399-0004.1995.tb03920.x
1995	Rousseaux et al.	3	86,0-88,0	10.1159/000134118
1996	Prigent et al.	1	84,9	Ninth French Cytogeneticists Congress, Paris (1996)
1997	Estop et al.	1	61,1	PMID: 9195156
1997	Menicke et al.	2	19-51	PMID: 9466181
1997	Pellestor et al.	2	33,3-44,4	10.1159/000134657
1997	Van Hummelen et al.	1	48,1	10.1086/515516
1998	Blanco et al.	1	45,1	10.1159/000015170
1998	Estop et al.	2	43,6-44,4	10.1159/000015177
1998	Martini et al.	1	44,3	10.1007/s004390050670
1998	Mercier et al.	1	56,7	10.1007/s004390050719
1999	Cifuentes et al.	1	49,6	10.1038/sj.ejhg.5200274
1999	Durak et al.	1	41,8	10.1034/j.1399-0004.1999.560206.x
1999	Estop et al.	1	27,4	10.1007/s004390050977
1999	Giltay et al.	1	51	10.1159/000015217
1999	Honda et al.	2	47,2-52,4	10.1007/s004399900161
1999	Van Assche et al.	1	29,1	10.1093/molehr/5.7.682
2001	Oliver-Bonnet et al.	1	30,5	10.1038/sj.ejhg.5200654
2002	Alves et al.	1	85,1	10.1038/sj.ejhg.5200835
2002	Cora et al.	4	48,1-52,7	PMID: 12399524
2002	Geneix et al.	1	19	10.1093/humrep/17.2.325
2002	Oliver-Bonnet et al.	2	39,2-41,6	10.1093/molehr/8.10.958

2002	Trappe et al.	1	45	10.1159/000068533
2003	Bacetti et al.	1	32,8	10.1093/humrep/deg460
2003	Escudero et al.	11	18,6-45,9	10.1016/s0015-0282(03)00252-8
2003	Lim et al.	1	49,9	10.1002/ajmg.a.10893
2003	Rives et al.	1	56,2	10.1007/s10038-003-0072-9
2004	Anton et al.	2	42,9-45	10.1016/s1472-6483(10)61774-9
2004	Morel et al.	2	54,68-62.84	10.1016/j.fertnstert.2003.07.034
2004	Oliver-Bonet et al.	2	40,8-44,6	10.1093/humrep/deh492
2006	Brugnon et al.	7	33-58,8	10.1093/humrep/dei401
2006	Midro et al.	1	34	10.1002/ajmg.a.31083
2006	Yakut et al.	4	37,1-51,3	10.1002/ajmg.a.31215
2007	Kekesi et al.	3	20,2-54	10.1016/j.fertnstert.2006.11.097
2007	Wiland et al.	2	34,4-34,8	10.2164/jandrol.106.000919
2008	Nishikawa et al.	4	40,7-54	10.1111/j.1365-2605.2007.00759.x
2008	Perrin et al.	1	54,3	10.1093/humrep/dem359
2008	Vozdova et al.	3	44,5-59,9	10.1093/humrep/dem345
2008	Wiland et al.	1	34,2	10.1002/pd.1899
2009	Vialard et al.	1	50,3	10.1016/s1472-6483(10)60133-2
2010	Perrin et al.	12	34,4-49,1	10.1111/j.1439-0272.2009.00951.x
2011	Cassuto et al.	6	37,1-52,6	10.1016/j.fertnstert.2011.07.1143
2011	Vozdova et al.	2	48,1-50,7	10.1016/j.fertnstert.2011.04.042
2012	Chelli et al.	6	46,4-67,4	10.1007/s10815-013-9959-3
2012	Godo et al.	8	30,2-50,7	10.1093/humrep/des431
2012	Kasikova et al.	2	49,5-55,4	10.1007/s10815-012-9767-1
2012	Mokanszki et al.	1	46,8	10.3109/19396368.2012.670868
2012	Vozdova et al.	5	31,9-50,7	10.1093/humrep/der445
2013	Ferfouri et al.	2	48,1-49,6	10.1093/molehr/gas048
2013	Olszewska et al.	10	34,3-62,4	10.1016/j.repbio.2013.06.002
2013	Rouen et al.	12	25,2-47,7	10.1093/humrep/det121
2013	Vozdova et al.	27	31,1-55,4	10.1007/s10815-012-9921-9
2014	Midro et al.	1	40,37	10.1038/jhg.2014.92
2016	Midro et al.	2	51,9-52.4	10.1007/s10815-015-0622-z
2017	Haapaniemi Kouru et al.	10	28-72	10.1016/j.rbmo.2017.02.013
2017	Rouen et al.	10	23,0-51,0	10.1016/j.rbmo.2017.06.017
2019	Zenagui et al.	7	33,9-43,9	10.1016/j.rbmo.2019.02.010
2020	Lei et al.	40	20,2-52,5	10.1007/s10815-020-02013-z
2022	Del Llano et al.	41	27,8-69,7	-

[1] DOI until precised otherwise.

Supplemental Table S8. Mix of probes used in spermFISH analysis.

Patient	Karyotype	Probes
P1	t(1;4)(p21;q13)	CEP 4 SA[1], Tel 1p SO[2] & Tel 4q SG[2]
P2	t(1;6)(q42;p22)	CEP 1 SO[1], CEP 6 SA[1] & Tel 1q SG[2]
P3	t(1;11)(p34;q24)	CEP 1 SO[1], CEP 11 SA[1] & Tel 1p SG[2]
P4	t(1;11)(q12;q24)	CEP 1 SO[1], CEP 11 SA[1] & Tel 1p SG[2]
P5	t(2;4)(q24;p15,.1)	CEP 2 SO[1], CEP 4 SA[1] & Tel 4p SG[2]
P6	t(2;7)(p25;p15.1)	CEP 7 SO[1], Tel 2p SA[2] & Tel 7p SG[2]
P7	t(2;10)(q21;q24)	CEP 10 SA[1], Tel 2p SG[2] & Tel 2q SO[2]
P8	t(2;12)(q14.2;q15)	CEP 2 SO[1], CEP 12 SG[1] & Tel 2q37.1-2q37.2 SA[3]
P9	t(3;11)(p24;p12)	CEP 11 SA[1], Tel 3p SG[2], Tel 3q SO[2]
P10	t(3;12)(q13.1;q13)	CEP 3 SA[1], Tel 3q SG[2] & Tel 12q SO[2]
P11	t(4;8)(q31.1;p21)	CEP 4 SA[1], CEP 8 SG[2] & Tel 4q SO[2]
P12	t(4;10)(q21;q24)	CEP 10 SA[1], Tel 4q SG[2] & Tel 10q SO[2]
P13	t(4;14)(p14;q32)	CEP 14[1] SA, Tel 4p SG[2] & Tel 14q SO[2]
P14	t(4;17)(p16.1;q21.3)	CEP 4 SA[1], Tel 4p SO[2] & Tel 17q SG[2]
P15	t(4;21)(q28;q21)	CEP 4 SA[1], Tel 4p SO[2], Tel 21q SG[2]
P16	t(5;18)(p15.1;q12.3)	CEP 18 SA[1], Tel 18q SO[2], Tel 5p SG[2]
P17	t(6;12)(p25;p13)	CEP 6 SA[1], CEP 12 SO[2] & Tel 6p SG[2]
P18	t(6;15)(p12;p13)	CEP 15 SA[1], CEP 6 SG[1] & Tel 15q SO[2]
P19	t(6;15)(q12;q21)	CEP 15 SA[1], CEP 6 SG[1] & Tel 15q SO[2]
P20	t(6;18)(p21.1;q23)	CEP 6 SA[1], Tel 6p SG[2] & Tel 18q SO[2]
P21	t(6;18)(p21.1;q23)	CEP 6 SA[2], Tel 6p SG[2] & Tel 18q SO[2]
P22	t(7;9)(p14;q21)	CEP 7 SA[1], CEP 9 SO[2] & Tel 7p SG[2]
P23	t(8;16)(p12;q23)	CEP 8 SA[1], CEP 16 SO[2] & Tel 8q SG[2]
P24	t(8;16)(p12;q23)	CEP 8 SA[1], CEP 16 SO[2] & Tel 8p SA[2]
P25	t(8;20)(p12;q12)	CEP 8 SA[1], CEP 20 SO[2] & Tel 8p SA[2]
P26	t(9;11)(p24;q23)	CEP 9 SA[1], CEP 11 SG[2] & Tel 11q SO[2]
P27	t(9;17)(p22;q23)	CEP17 SA[1], CEP 9 SO[2] & Tel 9p SG[2]
P28	t(9;17)(q22;q21)	CEP 9 SA[1], Tel 9q SG[2] & Tel 17q SO[2]
P29	t(10;17)(q11.2;q25)	CEP 10 SA[1], Tel 10q SO, Tel 17q SG[2]
P30	t(10;17)(p15;q12)	CEP 10 SA[1], Tel 10q SO, Tel 17q SG[2]
P31	t(13;15)(q32;q22)	CEP 15 SA[1], Tel 13q SG, Tel 15q SO[2]
P32	t(13;18)(q14;q22)	CEP 18 SA[1], Tel 18q SO[2], Tel 13q SG[2]
P33	t(14;17)(q22;p11.2)	CEP 17 SA[1], Tel 17p SG[2], Tel 14q SO[2]
P34	t(8;16)(p22;p12)	Tel 16p13.3 SG[3], Tel 16q24.1-24.2 SO[3] & CEP8 SA[1]
P35	t(2;13)(q33;q14)	Tel 2q35 SO[3], Tel 2p21 SA[3] & Tel 13q33.3 SG[3]
P36	t(8;19)(p23;p11)	Tel 8q24.21 SO[3], Tel 19p13.11 SA[3] & Tel 19q13.43 SG[3]
P37	t(12;15)(q14;q25)	Tel 12q24.21 SG[3], Tel 15q26.1 SO[3] & Tel 12p13.33 SA[3]
P38	t(8;20)(q24.3;q11)	Tel 8p23.2 SA[3], Tel 20p13 SO[3] & Tel 20q12 SG[3]
P39	t(4;5)(q35;q22)	Tel 5p13.2 SO[3], Tel 5q31 SG[3] & Tel 4q22.1 SA[3]
P40	t(1;2)(p22;p14)	Tel 1p36.33 SG[3], Tel 2p21 SO[3] & Tel2q35 SA[3]
P41	t(2;4)(p13;q27)	Tel 2p21 SG[3], Tel 4q34.1 SO[3] & Tel 2q35 SA[3]

[1] Vysis®, ABBOTT ; [2] CytoCell, Amplitech ; [3] Homemade probe.

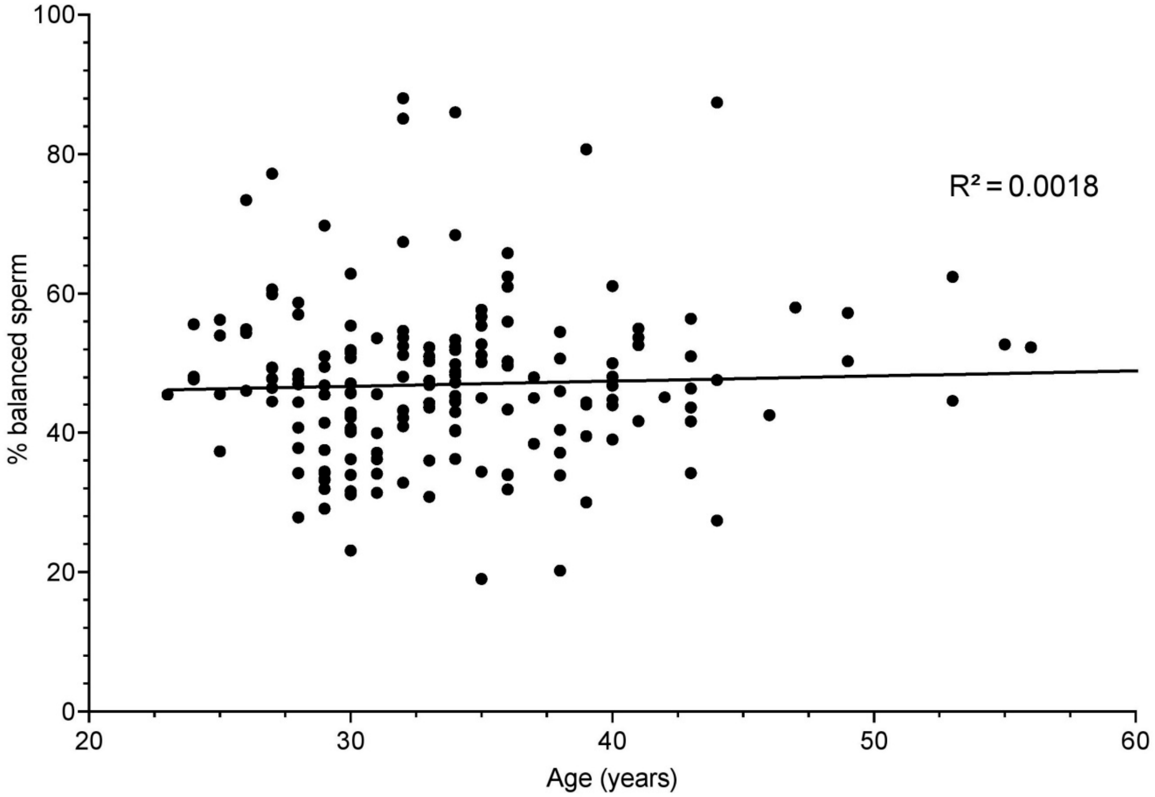

Figure S1. Balanced sperm rates are not correlated with patient age.
